# Serum exosomal hsa-circ-0004771 modulates the resistance of colorectal cancer to 5-fluorouracil via regulating miR-653/ZEB2 signaling pathway

**DOI:** 10.1186/s12935-023-03072-9

**Published:** 2023-10-16

**Authors:** Xiao-Xue Qiao, Hui-Bo Shi, Li Xiao

**Affiliations:** 1 The Third Clinical Medical College (School of Clinical Medicine), Fujian Medical University, Fuzhou, 350004 China; 2 Department of Oncology, Zhongshan Hospital Affiliated to Xiamen University, Xiamen, 361004 China; 3 Department of Institute of Organ Transplantation, Tongji Hospital, Tongji Medical College, Huazhong University of Science and Technology, Wuhan, 430030 China; 4 Department of Key Laboratory of Organ Transplantation, Ministry of Education, Tongji Hospital, Tongji Medical College, Huazhong University of Science and Technology, Wuhan, 430030 China; 5 Department of NHC Key Laboratory of Organ Transplantation, Tongji Hospital, Tongji Medical College, Huazhong University of Science and Technology, Wuhan, 430030 China; 6 Department of Key Laboratory of Organ Transplantation, Chinese Academy of Medical Sciences, Tongji Hospital, Tongji Medical College, Huazhong University of Science and Technology, Wuhan, 430030 China

**Keywords:** Colorectal cancer, 5-fluorouracil, Drug-resistance, Serum exosome, Hsa-circ-0004771/miR-653/ZEB2 axis

## Abstract

**Background:**

Drug resistance is a major obstacle causing chemotherapy failure, and enabling cancer progression. Exosome excreted by cancer cells is participated in cancer progression and chemoresistance, and can be used as an prognostic biomarker. Previous studies have revealed that serum exosomal hsa-circ-0004771 is over-expressed in colorectal cancer (CRC) sufferers and suggested it as a predictive biomarker for early diagnosis and prognosis of CRC. This work will to investigate the role and mechanism of serum exosomal hsa-circ-0004771 in mediating resistance to 5-fluorouracil (5-FU) in CRC.

**Methods:**

Serum and tissue samples were collected from 60 patients with CRC/ benign intestinal disease, and 60 healthy control. Exosomes were isolated and identified from serum samples and cell cultured media with TEM, WB, NTA, and flow cytometry. qRT-PCR and WB were performed to evaluate mRNA expressions of exosomal has-circ-0004771 and miR-653, and ZEB2 protein expression, respectively. Cell proliferation, migration, invasion, and apoptosis abilities were assessed with BrdU and colony formation assay, wound-healing assay, and flow cytometry, respectively.

**Results:**

Exosomal hsa-circ-0004771 was over-expressed in CRC serum and cell cultured media, while miR-653 was lower-expressed in CRC tissues and cells. Negative correlations existed between exosomal hsa-circ-0004771 in the patients^’^ serum/cell culture media and miR-653 in CRC tissues/cells, and between miR-653 and ZEB2 in CRC cells. Exosomal hsa-circ-0004771 in CRC cell cultured media was positively related to ZEB2 in CRC cells. MiR-653 was associated with poor prognosis of CRC patients, and its upregulation restrained CRC cell proliferation, migration and invasion, and stimulated apoptosis. Exosomal hsa-circ-0004771 was higher-expressed in 5-FU-resistant CRC serum and cell cultured media, miR-653 was downregulated and ZEB2 was overexpressed in 5-FU-resistant CRC cells. In vitro, exosomal hsa-circ-0004771 in cell cultured media may be involved in 5-FU-resistance by modulating miR-653/ZEB2 pathway.

**Conclusions:**

miR-653 plays as a tumour suppressor in CRC progression, and serum exosomal hsa-circ-0004771 may be a predictive biomarker for 5-FU-resistance in CRC patients, potentially through miR-653/ZEB2 axis.

**Supplementary Information:**

The online version contains supplementary material available at 10.1186/s12935-023-03072-9.

## Background

Due to the similar characteristics and therapeutic strategies for treating colon cancer and rectal cancer, they are collectively named as colorectal cancer (CRC). CRC is the third most prevalent malignancy and the second most leading reason of cancer-associated death around the world [1]. Globally, more than 1.8 million new CRC cases were diagnosed and about 862000 CRC patient death were announced in 2018 [1], and by 2030, it is predicted that 2.2 million new cases and 1.1 million CRC deaths will be produced worldwide [2]. While the continuous progress in surgical treatments, radiotherapy and chemotherapy, and the availability of molecular targeted therapy and immunotherapy, has ameliorated the overall survival and prognosis of CRC sufferers, the overall prognosis of CRC is still not satisfactory [3]. The 5 year survival ratio of early CRC sufferers is more than 90%, but the median survival duration of advanced CRC patients is less than 2 years [4]. With improved economic status, changes in the life style and diet, and altered environment, the morbidity and mortality of CRC patients has been increasing year by year and often diagnosed at a younger age [5, 6]. Because most of the CRC patients do not show any symptoms at an early stage, they usually present with an advanced disease at diagnosis, leading to the loss of opportunity for surgical treatment, accompanying by a poor prognosis of patients with liver metastasis or lung metastasis [7, 8]. Therefore, chemotherapy exerts an momentous action in treating CRC, especially metastatic CRC. The tumor growth, metastasis and recurrence can be controlled by chemotherapy [9]. 5-Fluorouracil (5-FU) as the first-line treatment for CRC [10], can induce alterations in RNA processing/function and promotes severe DNA damage resulting in apoptosis [11, 12]. Despite the benefits of 5-FU in treating CRC, the 5 year survival ratio is still stagnant [13]. The innate or acquired resistance to 5-FU is the cardinal factor affecting the therapeutic response of the patients [14], therefore, it is critical to study the mechanisms promoting 5-FU resistance in order to more effectively manage CRC therapy.

Evidence suggests that exosomes from CRC cells are closely associated with tumorigenesis, distant metastasis, and chemoresistance [15]. It is well known that exosomes are vesicles with a phospholipid bilayer membrane with a diameter from 30 to 150 nm, and exocytosed by the cells. Almost all types of cells in the human body can secrete exosomes under normal physiological and pathological conditions, and all types of cultured cells can secrete exosomes as well [16–18]. Exosomes are widely found in body fluids, including blood, lymph, saliva, urine, semen, breast milk, ascitic fluid, amniotic fluid, cerebrospinal fluid, tear, nasal mucus, broncho-alveolar lavage fluid and so on [16, 19]. Exosomes are rich in a variety of bioactive substances, including nucleic acids (miRNA, lncRNA, circRNA, mRNA, tRNA), proteins (e.g., fusion proteins, membrane transporters, heat shock proteins), lipids (e.g., cholesterol, phospholipids), etc. The content carried by the exosomes depend on the functional state of the cells from which they originate, and exosomes can be involved in multiple biological processes, such as intercellular communication, cell proliferation, cell migration, cell differentiation, angiogenesis, and immunomodulation etc. [20–22]. A previous study suggested that the “unique” membrane structure of exosomes enables the exosomes to be internalized in a stable state, while protecting them from various micro-environmental factors in the extra-membranous milieu [23]. Hence, certain active substances found in the exosomes in body fluids may serve as biomarkers for cancer. For instance, hsa-circ-0004771 found in the exosomes isolated from the serum of CRC patients was elevated, which could be used as a biomarker for early diagnosis and prognosis of CRC [24]. However, whether hsa-circ-0004771 in serum derived exosomes is involved in mediating 5-FU resistance in CRC patients has not been reported so far.

Previous studies have reported the presence of targeted binding sites between hsa-circ-0004771 and miR-653 [25]. miR-653 was down-regulated in cancers such as breast cancer [25], and overexpression of miR-653 played tumor suppressive role, which could attenuate cell proliferation, invasion, metastasis, and tumor growth, and induce apoptosis [25–28]. However, the expression and function of miR-653 in CRC have not been investigated and whether miR-653 is involved in mediating 5-FU resistance is unclear. Furthermore, a targeted binding site between miR-653 and zinc finger E-box binding homeobox 2 (ZEB2) has also been validated, and hsa-circ-0004771 is reported to modulate the miR-653/ZEB2 axis to promote breast cancer progression [25]. Similarly, it has been shown that ZEB2 is over-expressed in CRC tissue samples and cell lines, and its high expression has been reported to promote the malignant biological behaviors of CRC, such as boosting proliferation, metastasis, epithelial-mesenchymal transition and accelerating tumor growth and tumor formation [29]. Also, the up-regulation of ZEB2 enhanced the resistance of CRC patients to 5-FU [30]. Hence, the degree of drug resistance of CRC cells to 5-FU treatment may be suppressed by modulating ZEB2 expression in CRC cells or tissues. Nevertheless, it is unclear whether the serum derived exosomes containing hsa-circ-0004771 could also regulate miR-653/ZEB2 axis to regulate the resistance of CRC cells to 5-FU. To answer this question, we carried out a series of experiments: (1) In the clinical setting, we isolated and identified exosomes from CRC patient serum; determined the correlation between the serum derived exosomal hsa-circ-0004771 and 5-FU resistance in CRC, determined the correlation between the serum derived exosomal hsa-circ-0004771 and miR-653 expression in CRC and the clinical significance of miR-653 in CRC; (2) in vitro experiments to determine the role of miR-653 in the malignant biological phenotype of CRC, determining whether the exosome-delivered hsa-circ-0004771 from cell culture media could mediate 5-FU resistance by regulating miR-653/ZEB2 axis in CRC.

## Materials and methods

### Patients and clinical samples

60 patients with colorectal adenocarcinoma (accounting for about three-quarters of CRC), 60 colorectal adenoma patients [benign intestinal disease (BID)], and 60 healthy volunteers who participated in physical examination, including enteroscheocele (healthy control (HC)], from Sep 2018 to Sep 2019 in our hospital, were enrolled in this study. CRC and BID patients were initially confirmed by histopathological analysis of the resected tissues from electronic colonoscopy. The criteria for selecting the subjects were as follows: (A) For colorectal adenocarcinoma patients: (1) all cases were histopathologically diagnosed with colorectal adenocarcinoma using enteroscopy; (2) No CRCs-related surgery, chemoradiotherapy, and other tumor-related treatments were performed before admission; (3) Were treated conservatively with 5-FU alone; (4) clinical case data were complete; (5) absence of other tumors; (6) absence of other intestinal diseases; (7) absence of heart, liver, kidney, and other organ dysfunctions; (8) absence of infection or pregnancy. (B) For colorectal adenoma patients: (1) be histopathologically diagnosed with colorectal adenoma by enteroscopy; (2) absence of history of tumors; (3) absence of other intestinal diseases; 4) absence of heart, liver, kidney, and other organ dysfunctions. (3) For healthy volunteers: (1) Absence of abnormality in the intestinal tract after endoscopy; (2) Absence of history of tumors; (3) Absence of heart, liver, kidney, and other organ dysfunctions. For BID and HC groups, the serum samples (5 ml of peripheral venous blood under empty stomach) and fresh tissues were collected within 24 h of admission, and for the CRC group, the serum samples were collected within 24 h of admission and after the end of chemotherapy with 5-FU, while fresh tissues were acquired within 24 h of admission. The acquired serum samples and fresh tissues were immediately stored in liquid nitrogen and then stored in the – 80 ℃ refrigerator. For all the above groups, the basic clinical information including age, sex, body mass index (BMI), the history of smoking, drinking, and familial inheritance were collected and analyzed (Table 1). In addition, for the CRC group, the basic clinical data including tumor location, tumor size, tumor differentiation degree, tumor invasion depth, lymphatic metastasis, TNM staging, and 5 years survival rate (follow up data) were also collected (Table 2). The research protocol was approved by the ethics committee of our hospital, and informed consent was obtained from the family of the patient before the initiation of study.

**Table 1:** Comparison of baseline data between groups

Parameters	Groups			P values
	CRCs (n = 60)	BIDs (n = 60)	HCs (n = 60)	
Age (x ± s, years old)	61.4 ± 8.8	59.2 ± 14.3	58.5 ± 13.2	> 0.05
Genders				
Male	38	36	39	> 0.05
Female	22	24	21	
BMI (x ± s, kg/m^2^)	23.1 ± 2.5	23.3 ± 2.7	22.3 ± 3.2	> 0.05
History of smoking				
Yes	34	32	27	> 0.05
No	26	28	33	
History of drinking				
Yes	35	33	29	> 0.05
No	25	27	31	
History of familial inheritance				
Yes	22	20	18	> 0.05
No	38	40	42	

*CRCs* colorectal cancer, *BIDs* benign intestinal diseases, *HCs* healthy controls, *BMI* body mass index

**Table 2:** Association between miR-653 expression in colorectal cancer tissues and the clinico-pathological features of colorectal cancer patients

Characteristics	Number of cases	Expression of miR-653		P value
		Low (n = 30)	High (n = 30)	
Gender				
Male	38	20	18	P > 0.05
Female	22	10	12	
Age, years				
< 60	24	11	13	P > 0.05
≥ 60	36	19	17	
Tumor location				
Colon	31	15	16	P > 0.05
Rectum	29	14	15	
Tumor size				
< 5 cm	35	25	10	P < 0.05
≥ 5 cm	25	18	7	
Differentiation degree of tumor				
Low	15	11	4	P < 0.05
Middle	20	12	8	
High	25	8	17	
Infiltration depth				
T1 + T2	27	10	17	P < 0.05
T3 + T4	43	28	15	
Lymphatic metastasis				
N0 + N1	32	12	20	P < 0.05
N2 + N3	28	20	8	
TNM stage				
I-II	33	12	21	P < 0.05
III-IV	27	18	9	
Five years survival rate				
Yes	21	8	13	P < 0.05
No	39	27	12	

### Cell lines and cell culture

The normal human intestinal epithelium cell line (NCM460 (*#*GD-C6818571)) and human CRC cell lines (SW480 (*#*TCHu 86), HT29 (*#*TCHu 103), DLD-1 (*#*TCHu 134), SW620 (*#*TCHu 101), and HCT116 (*#*TCHu 99)) were purchased from the Cell Bank of Chinese Academy of Sciences (Shanghai, China). All the cells were tested and authenticated by STR profiling followed by cell culture and incubation as previously published [31]. The cell culture media were collected for subsequent experiments.

### Development of 5-FU-resistant SW620 and HCT116 cells

5**-**FU-resistant SW620 cells (SW620-R) were generated by continuously exposing the cells to an increasing concentration gradient of 5-FU (#F6627, 10–200 μM, Sigma-Aldrich) and cultured in Dulbecco’s Modified Eagle’s Medium (DMEM, *#*12800017, Invitrogen, Carlsbad, CA, USA) containing 10% fetal bovine serum (FBS, #26400044, Invitrogen, USA), 1% penicillin/streptomycin (*#*15070063, Gibco, BRL) at 37℃ in a humidified incubator with 5% CO2 based on the recent report in 2020 [32]. 5-FU-resistant HCT116 cells (HCT116-R) were established by treating them with gradually increasing concentrations of 5-FU (#F6627, 5 μM to 50 μM, Sigma-Aldrich) for 9 months and were maintained in Iscove’s modified Dulbecco’s medium (IMDM, *#*12440–053, Invitrogen, Carlsbad, CA, USA) with 10% FBS (#26400044, Invitrogen, USA) and 1% penicillin and streptomycin (*#*15070063, Gibco, BRL), in a humidified incubator (5% CO2) at 37℃, as previously described [33]. In addition the cell culture media was collected for follow-up tests.

### Cell transfection

For overexpression of miR-653, miR-653 mimic (target sequence: 5′-UUGAAACAAUCUCUACUGAACC-3′) and its negative control (NC) miR (miR-NC) (target sequence: 5′-GCCAGCCCUGUAAGUCCCGCAU-3′) were designed and synthesized by GenePharma (Shanghai, China). For suppression of hsa-circ-0004771 expression, 50 nM of specific small interfering RNA (si-RNA) (hsa-circ-0004771 siRNA/si-hsa-circ-0004771, target sequence: 5′-GACAGACGGAAGTGTTTGGAT-3′; and si-circRNA-NC/siNC, target sequence: 5′-AAGCCGGAGCTTCGTGGAATC-3′), were designed and synthesized by RiboBio (Guangzhou, China). For transfection, miR-653 mimic, miR-NC, si-hsa-circ-0004771, siNC, si-hsa-circ-0004771 + miR-653 mimic were transfected into SW620 and HCT116 cells utilizing Lipofectamine 2000 Transfection reagent (#11668019, Invitrogen, USA), in accordance with the manufacturer’s instructions, at 37 ℃ for 48 h. The transfection efficiency was measured by qRT-PCR and the cell culture media was collected for further experiments.

### Isolation and identification of exosomes

Exosomes were isolated form all clinical serum samples and cell culture media of cell lines, as described previously [24]. For the identification of exosomes in human serum samples, transmission electron microscopy (TEM) analysis was conducted and the morphology of exosomes in serum samples and cell culture media was detected, as described previously [24]. Western blot (WB) was performed to assess the expression of exosome surface marker protein CD63 and TSG101 (calnexin was used as an internal control, which is an integrin expressed on endoplasmic reticulum and not expressed in exosomes) in clinical serum samples and cell culture media, as described previously [24]. For identification of exosomes in serum samples, nanoparticle tracking analysis (NTA) was used, wherein the diameter distribution was determined and the density of isolated exosomes was also measured according to the previous study [34]. Flow cytometry was used for measuring the proportion of exosomes with the positive surface marker molecules TSG101 and CD63, as per previously published literature [35].

### Quantitative real-time PCR (qRT-PCR)

Total RNA was extracted from exosomes in serum samples and cell culture media after treating them with TRIzol reagent (*#*15596026, Invitrogen, Carlsbad, CA, USA). Using the manufacturer’s instructions, cDNA was synthesized with a reverse transcription kit (*#*4368814, Invitrogen, USA) from mRNA by qRT-PCR, and miRNA was reversely transcribed into cDNAs with a miRNA-specific stem-loop reverse-transcription primer (*#*4427975, Ribobio, Guangzhou, China). For the assessment of hsa-circ-0004771 and ZEB2 mRNA expression, GAPDH was used as a reference gene, while human U6 snoRNA was applied as the internal control for the evaluation of hsa-miR-653 mRNA level. All primers were synthesized by RiboBio (Guangzhou, China) using the following sequences: hsa_circ_0004771: forward, 5′-TCCGGATGACATCAGAGCTAC-3′ and reverse, 5′-TCAAGTGTGCATCTTCTGGCT-3′; ZEB2: forward, 5′-CAAGAGGCGCAAACAAGC-3′ and reverse, 5′-GGTTGGCAATACCGTCATCC-3′; GAPDH: forward, 5′-GCACCGTCAAGCTGAGAAC-3′ and reverse, 5′-TGGTGAAGACGCCAGTGGA-3′; hsa-miR-653: forward, 5′-ACCAGCTTC AAA CAAGTTCACTG-3′ and reverse, 5′-GCTTCCATCTTATCA TTCTTGCA-3′; U6: forward, 5′-CTCGCTTCGGCAGCACATATACTA-3′ and reverse, 5′-ACGAAT TTGCGTGTCATCCTTGCG-3′. The qRT-PCR was conducted under the following optimized conditions: initial denaturation at 95 ℃ for 10 min, followed by 40 cycles of denaturation at 95 ℃ for 10 s, annealing at 60 ℃ for 15 s, and extension at 72 ℃ for 20 s. A final extension step was performed at 72 ℃ for 5 min to ensure complete extension of the amplified DNA fragments. The 2^−△△Ct^ method was adopted to calculate the relative fold change, with a fold change of 2.0 being considered as significant. All PCR assays were performed in triplicate.

### Western blotting (WB)

In order to determine the protein expression of ZEB2 in CRC cells, WB was performed as previously described [36]. For quantification, anti-ZEB2 primary antibody (#70512, 1:1000; CST, USA) was used and GAPDH (#5174, 1:2000; Santa Cruz Biotechnology, USA) was used as the internal control.

### Pearson correlation coefficient analysis

The Pearson correlation coefficient analysis was performed to evaluate the correlation in expression between hsa_circ_001653 and miR-653/ZEB2 (Table 3).

**Table 3:** Spearman correlation coefficient analysis

Gene names	Sources	Gene names	Sources
hsa-circ-0004771	Patients’ serum exosomes (Before any treatments)	miR-653	CRC tissue specimens (Before any treatments)
hsa-circ-0004771	Exosomes in SW620 cell culture media	miR-653	SW620 cells
hsa-circ-0004771	Exosomes in HCT116 cell culture media	miR-653	HCT116 cells
hsa-circ-0004771	Exosomes in SW620 cell culture media	ZEB2 mRNA	SW620 cells
hsa-circ-0004771	Exosomes in HCT116 cell culture media	ZEB2 mRNA	HCT116 cells
hsa-circ-0004771	Exosomes in cell culture media from SW620 cells which transfection with si-hsa-circ-0004771	miR-653	SW620 cells which transfection with si-hsa-circ-0004771
hsa-circ-0004771	Exosomes in cell culture media from HCT116 cells which transfection with si-hsa-circ-0004771	miR-653	HCT116 cells which transfection with si-hsa-circ-0004771
hsa-circ-0004771	Exosomes in cell culture media from SW620 cells which transfection with si-hsa-circ-0004771	ZEB2 mRNA	SW620 cells which transfection with si-hsa-circ-0004771
hsa-circ-0004771	Exosomes in cell culture media from HCT116 cells which transfection with si-hsa-circ-0004771	ZEB2 mRNA	HCT116 cells which transfection with si-hsa-circ-0004771

*CRC* colorectal cancer, *ZEB2* zinc finger E-box binding homeobox 2, *si* small interfering

### BrdU proliferation assay

To measure the impact of miR-653 overexpression on CRC cell proliferation, the BrdU proliferation assay was performed [37]. Briefly, SW620 and HCT116 cells transfected with miR-NC and miR-653 mimic were seeded in 96-well plates followed by incubation for 24 h. Following this, the thymidine analog 5-bromo-2′-deoxyuridine (BrdU, *#*M6348, Abmole Bioscience Inc. USA) was added to each well for 24 h. The cell proliferation was determined by a BrdU cell proliferation ELISA kit (#CBA-251, Abcam, UK) and the absorbance values were recorded under the guidelines of the manufacturer. The experiment was repeated thrice.

### Colony formation assay

Similar to the BrdU proliferation assay, colony formation assay was performed to detect the colony forming ability of SW620 and HCT116 cells when transfected with miR-NC and miR-653 mimic, as previously described [38]. In brief, the cells were cultured in six-well plates with 500 cells per well and incubated for 15 days in DMEM(*#*11995–065, Life Technologies, Gibco^®^) containing 10% FBS (*#*F6178, Sigma-Aldrich; Merck KGaA) at 37℃ in an incubator (Thermo Fisher Scientific, USA), the media was replaced every 3–5 days. Then, the colonies were fixed with formalin and then stained with 0.1% crystal violet (*#*C3886, Sigma Aldrich) for 10 min, both at room temperature, and counted under a microscope. The assay was repeated thrice.

### Transwell assays

Transwell assays were carried out to measure the effect of miR-653 overexpression on the migration and invasion capacities of cells using Boyden chamber assay (8 μm pore). The specific steps were as described in the previous study [39]. The assay was repeated thrice.

### Flow cytometry

After transfection for 72 h, the apoptotic rates of SW620 and HCT116 cells transfected with miR-NC and miR-653 mimic, were determined by Annexin-V-fluorescein isothiocyanate (VFITC)/propidium iodide (PI) apoptosis detection kit (*#*M3021, Carlsbad, Calif, USA), according to the manufacturer’s guideline and analyzed with flow cytometry (BD FACSCanto, USA). The procedure was as described in the previous study [37]. The experiment was repeated thrice.

### Statistical analysis

Statistical analysis was performed using SPSS software (version 20) and the results were depicted as mean ± standard deviation (SD), number (n), and rate (%). The comparison of quantitative data between two groups were conducted with independent-sample t-test and Student’s t-test, and among multiple groups were made with one-way analysis of variance (ANOVA). The percentage/rate data between groups were evaluated with X2 test, non-parametric Mann–Whitney U test, and Fisher's exact test. Significance levels were set at P < 0.05.

## Results

### Exosomes were successfully isolated and identified from clinical serum samples

**Fig. 1:**
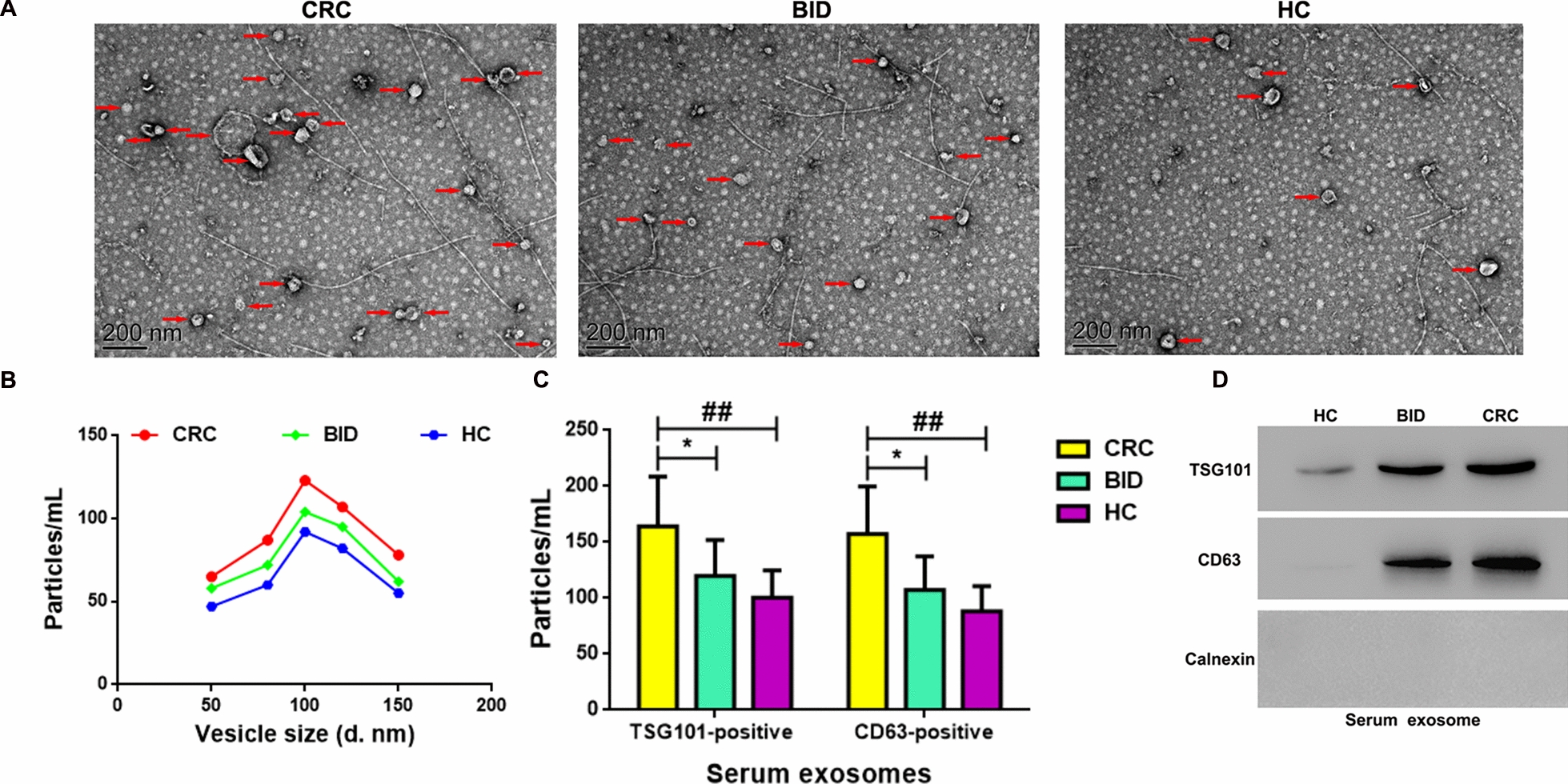
Isolation and identification of exosomes from clinical CRC serum samples. **A** The morphology of exosomes from clinical serum specimens identified by transmission electron microscopy (TEM). Scale bar: 200 nm; magnification: 30000 × ; red arrow: pointing to exosomes. **B** The diameter distribution of exosomes determined by nanoparticle tracking analysis (NTA). **C** The proportion of exosomes marked with positive TSG101/CD63 determined by flow cytometry. **D** The protein expression of TSG101 and CD63 (exosome surface markers) measured by western blotting (WB). *CRC* colorectal cancer, *BID* benign intestinal disease, *HC* healthy control. * (BID vs CRC), P < 0.05; ## (HC vs CRC), P < 0.01

Exosomes were isolated and identified from clinical serum samples by observing the morphology with TEM, measuring the diameter distribution and density with NTA, and detecting the expression of exosomal surface markers TSG101 and CD63 with flow cytometry and WB. The TEM outcomes showed that serum derived exosomes from CRC patients (n = 60), BID patients (n = 60), and HC (n = 60) were mainly round or round-like vesicles, and the number of exosomes from each group were: CRC > BID > HC (Fig. 1A). The NTA results displayed that the diameter of serum derived exosomes in the three groups were all between 50 to 150 nm, and the peak value of concentration of exosomes was at 100 nm, which met the diameter distribution standards of common exosomes in the human serum. Moreover, the relative concentration of exosomes in the three groups were: CRC > BID > HC (Fig. 1B). Besides, the ratios of TSG101 and CD63 positive exosomes in the serum from CRCs group were higher than in BID (P < 0.05) and HC group (P < 0.01) (Fig. 1C). CD63 and TSG101 were expressed in serum derived exosomes from all three groups, however calnexin was not expressed. The expression levels of CD63 and TSG101 in serum derived exosomes from CRC patients were significantly higher than that in BID (P < 0.05) and HC groups (P < 0.01) (Fig. 1D).

### The expression of hsa-circ-0004771 in serum derived exosomes from CRC patients was up-regulated and associated with 5-FU-resistance

**Fig. 2:**
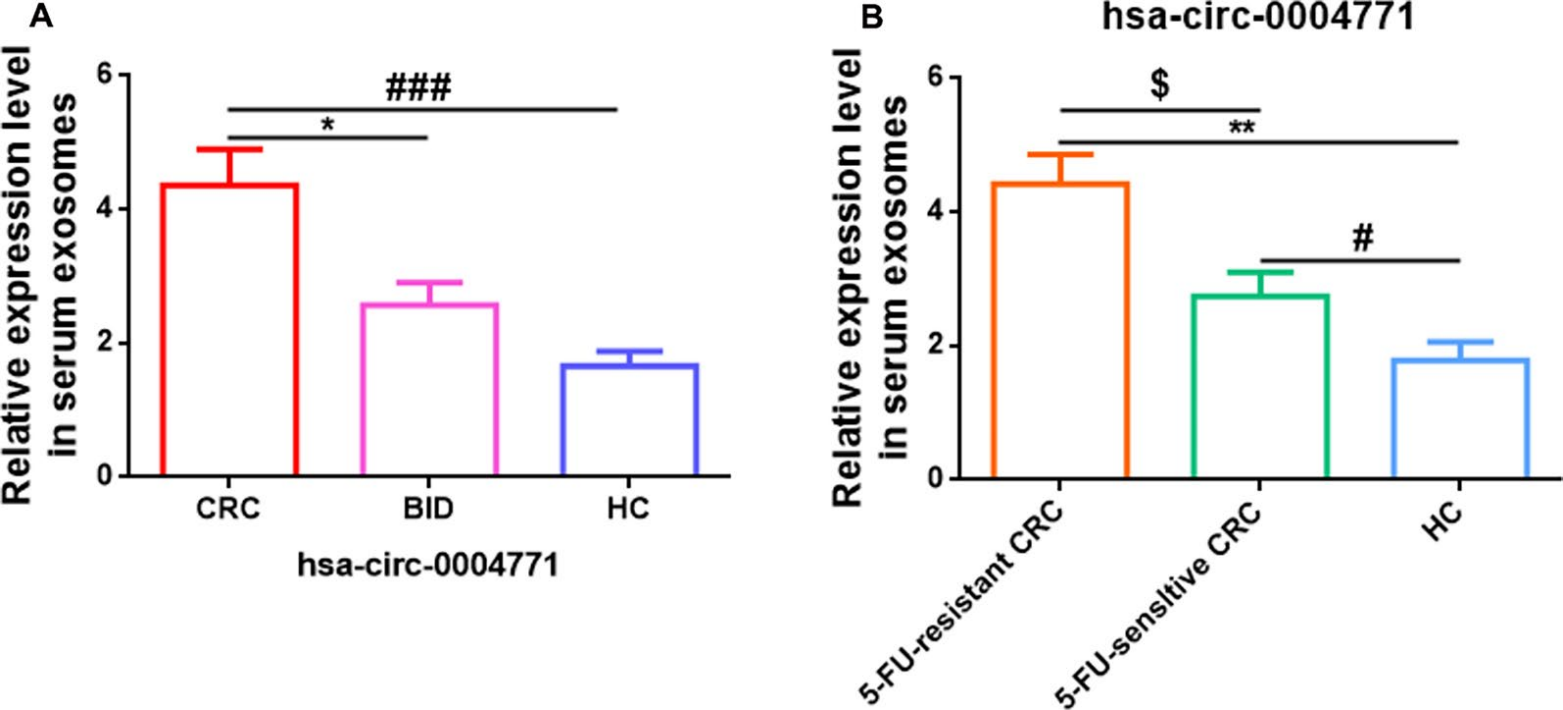
Hsa-circ-0004771 was up-regulated in serum exosomes and associated with 5-FU resistance in CRC patients. **A** qRT-PCR showing the relative expression level of hsa-circ-0004771 in serum exosomes from CRC (pre-treatment) and BID patients, and HC cases (* (CRC vs BID), P < 0.05; ### (HC vs CRC), P < 0.001). **B** qRT-PCR showing the relative expression level of hsa-circ-0004771 in serum exosomes from 5-FU resistant and sensitive CRC (after finish of chemotherapy with 5-FU) patients, and HC cases. $ (5-FU-resistant CRC vs 5-FU-sensitive CRC), P < 0.05; ** (5-FU-resistant CRC vs HC), P < 0.01; # (5-FU-sensitive CRC vs HC), P < 0.05. *CRC* colorectal cancer, *BID* benign intestinal disease, *HC* healthy control; 5-FU: 5-fluorouracil

Firstly, in order to ensure the uniformity between groups, statistical analysis was performed on the basic clinical data including age, sex, BMI, the history of smoking, drinking, and familial inheritance, and the results showed no statistical difference between the groups (P > 0.05) (Table. 1). Afterwards, exosome-delivered hsa-circ-0004771 levels were detected in clinical serum samples without any treatments by qRT-PCR. As shown in Fig. 2A, serum derived exosomal hsa-circ-0004771 in the BID group was higher than in the HC group, but there was no statistical difference (P > 0.05). Moreover, the serum derived exosomal hsa-circ-0004771 in the CRC group was upregulated compared to the HC (P < 0.001) and BID (P < 0.05) groups. In the CRC group, the patients were categorized into 5-FU-sensitive group and 5-FU-resistant group based on the therapeutic response to 5-FU. The efficacy of 5-FU can be assessed by monitoring the disease progression in patients. Simply, if the tumor exhibits significant shrinkage or disappearance following 5-FU treatment, it can be considered as sensitive to 5-FU. Conversely, if the tumor continues to grow or shows no substantial change after treatment, there may be evidence of drug resistance. Specifically, the chemotherapeutic effectiveness was evaluated using the World Health Organization (WHO) criteria for solid tumors based on computed tomography (CT) imaging. The criteria for judgment were as follows: Complete remission (CR): complete disappearance of all visible tumor lesions; partial remission (PR): > 50% reduction in the product of the maximum tumor diameter and its maximum vertical transverse diameter, with no amplification of other lesions and no appearance of new lesions; stability (SD): < 50% reduction in the product of the two diameters of the tumor lesions or an enlargement not exceeding 25%, with no appearance of new lesions; progression (PD): > 25% increase in the product of the two diameters of the tumor lesions or the appearance of new lesions. CR and PR were considered as indicative of sensitivity, while SD and PD were considered as indicative of resistance. The qRT-PCR results showed that compared with the serum derived exosomal hsa-circ-0004771 in the HC group which without any treatments, the expressions of serum derived exosomal hsa-circ-0004771 in 5-FU-sensltive (P < 0.05) and 5-FU-resistant CRC group (P < 0.01) were upregulated. Meanwhile, the serum derived exosomal hsa-circ-0004771 in the 5-FU-resistant CRC group was upregulated as compared to that in the 5-FU-sensltive group (P < 0.05) (Fig. 2B).

### miR-653 was downregulated in CRC tissues, and negatively correlated with serum derived exosomal hsa-circ-0004771 in CRC patients, and correlated with the severity and poor prognosis of CRC patients

**Fig. 3:**
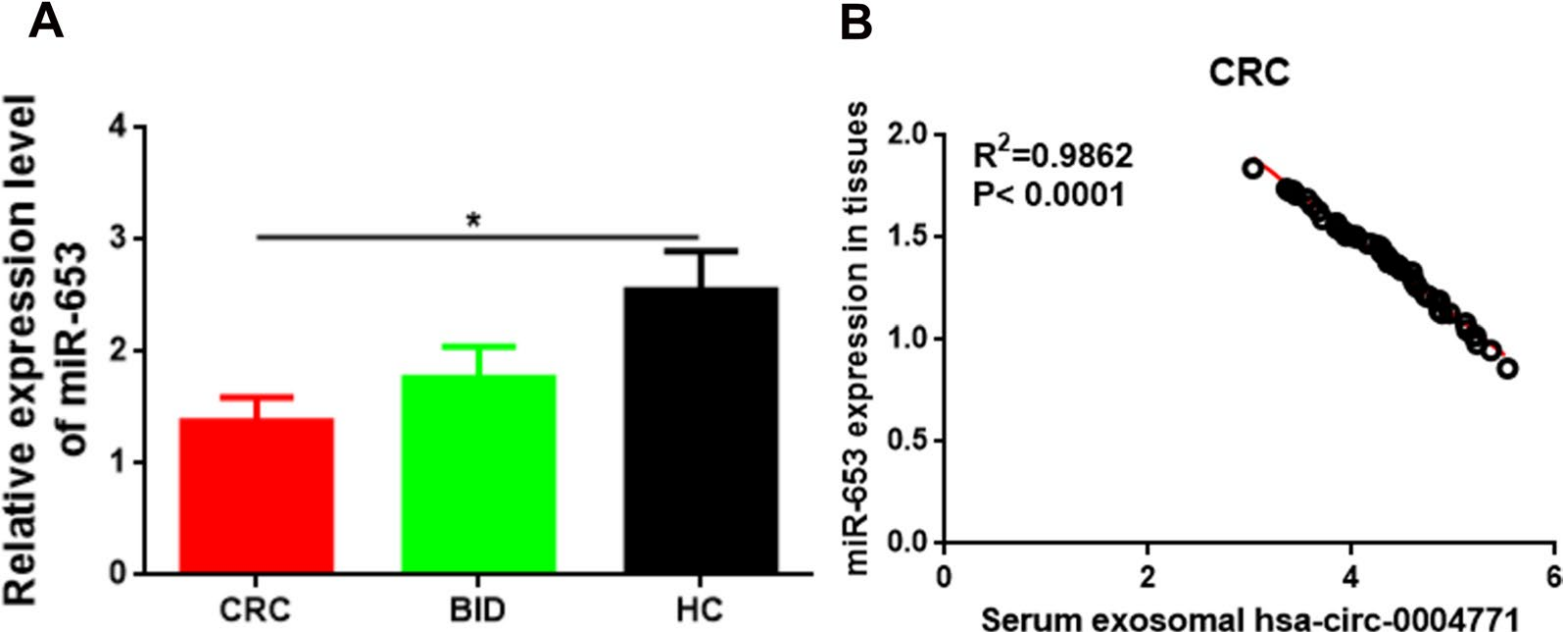
miR-653 was down-regulated in human CRC tissues and inversely correlated with serum exosomal hsa-circ-0004771 expression in CRC patients. **A** qRT-PCR showing the relative expression level of miR-653 in CRC and BID patients, and HC cases. (* (CRC vs HC), P < 0.05). **B** Spearman correlation analysis showing the relationship between miR-653 expression in CRC tissues and serum exosomal hsa-circ-0004771 expression in CRC patients. *CRC* colorectal cancer, *BID* benign intestinal disease, *HC* healthy control

As could be seen from Fig. 3A, qRT-PCR data showed that the expression of miR-653 was higher the BID (P > 0.05) and CRC groups (P < 0.05), as compared to the HC group. Spearman correlation coefficient analysis revealed that miR-653 expression in CRC tissues was negatively correlated to the expression of hsa-circ-0004771 in serum derived exosomes (P < 0.05) before any treatment (Fig. 3B). Interestingly, the expression of miR-653 in CRC tissues was negatively correlated to the severity and prognosis of CRC patients. In conclusion, there was no correlation between miR-653 level in CRC tissues and the sex/age/tumor location (P > 0.05). Moreover, lower expression of miR-653 was positively associated with higher severity and poor prognosis of CRC patients (P < 0.05 or P < 0.01) (Table. 2).

### miR-653 was downregulated in CRC cells, and overexpression of miR-653 repressed cell proliferation, migration, invasion, and promoted apoptosis

**Fig. 4:**
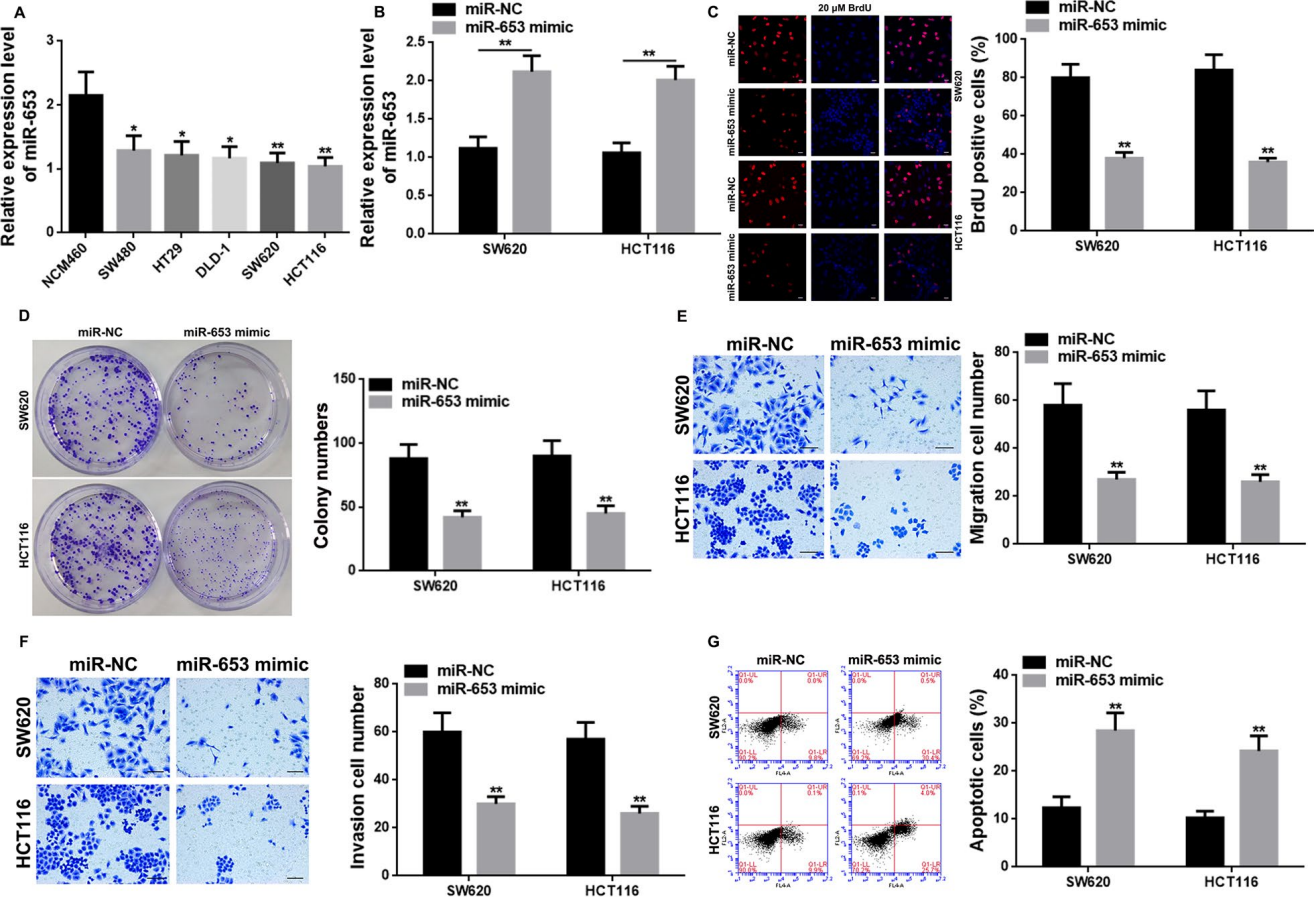
Effect of miR-653 knockdown and overexpression on CRC cell proliferation, migration, invasion, and apoptosis. **A** qR-PCR showing the relative expression level of miR-653 in CRC cells (* vs NCM460, P < 0.05; ** vs NCM460, P < 0.01). **B** qRT-PCR data showing the relative expression level of miR-653 in SW620 and HCT116 cells transfected with miR-NC and miR-653 mimic. **C** BrdU assay data showing the overexpression of miR-653 to reduce the percentage of BrdU positive cells. Red, SW620 and HCT116 cells labeled with BrdU; blue, nuclei counterstained by BrdU. Scale bar: 10 μm. **D** Colony formation assay showing the overexpression of miR-653 to weaken the clongenic ability. **E**, **F** Transwell assay showing the overexpression of miR-653 to suppress cell migration and invasion abilities. Scale bar: 8 μm. **G** Flow cytometry showing the overexpression of miR-653 to increase the percentage of apoptotic cells (** vs miR-NC, P < 0.01. *NC* negative control)

qRT-PCR was used to determine the expression of miR-653 in the normal human intestinal epithelial cell line NCM460, human CRC cell lines SW480, HT29, DLD-1, SW620, and HCT116. The results showed that the expression of miR-653 in NCM460 cells was higher than its expression in all the other CRC cell lines (P < 0.05 or P < 0.01), and the lowest expression was found in SW620 and HCT116 cells (Fig. 4A). Therefore, SW620 and HCT116 cells were used for subsequent experiments. To confirm the function of miR-653 in CRC, we generated SW620 and HCT116 cells overexpressing miR-653, by transfecting with miR-653 mimic (Fig. 4B). Thereafter, BrdU assay; colony formation assay; wound-healing assay; and flow cytometry assays were performed for assessing the role of miR-653 overexpression on cell proliferation, migration and invasion, and apoptosis, respectively. The results showed that the cells overexpressing miR-653 exhibited attenuated cel proliferation (P < 0.01) (Fig. 4C, D), reduced migration and invasion capacities (P < 0.01) (Fig. 4E, F), and increased apoptosis (P < 0.01) (Fig. 4G), as compared to cells transfected with the negative control (Fig. 4G).

### Exosome-delivered hsa-circ-0004771 in CRC cell culture media could regulate miR-653/ZEB2 pathway

**Fig. 5:**
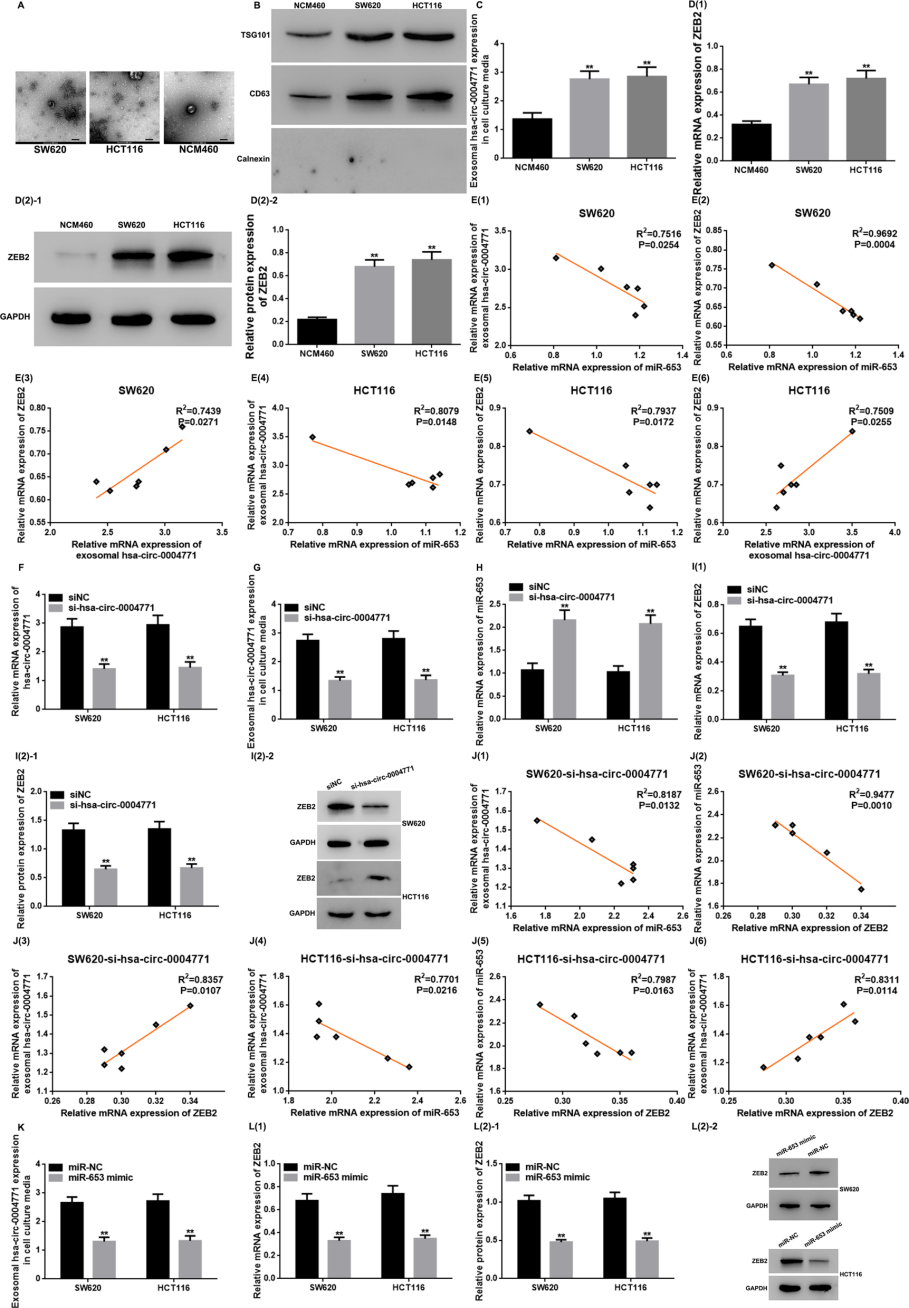
Exosomal hsa-circ-0004771 in CRC cell culture media regulates miR-653/ZEB2 axis. **A** The morphology of exosomes from cell culture media was identified by TEM. Scale bar: 200 nm; magnification: 30000 × . **B** The protein expression of TSG101 and CD63 (exosome surface markers) measured by WB. **C** qRT-PCR data showing upregulation of exosomal hsa-circ-0004771 in CRC cell culture media. (** vs NCM460, P < 0.01). **D** qRT-PCR and WB showing the upregulation of the relative mRNA and protein expression of ZEB2 in CRC cells, respectively. (** vs NCM460, P < 0.01). **E** Spearman correlation analysis showing the association between exosomal hsa-circ-0004771 expression in CRC cell culture media and miR-653/ZEB2 expression in CRC cells, and between miR-653 and ZEB2 in CRC cells. **F**–**H** qRT-PCR data showing decrease in hsa-circ-0004771 in CRC cells and exosomal hsa-circ-0004771 in cell culture media, and an increase of miR-653 in CRC cells after transfection with hsa-circ-0004771 (** vs siNC, P < 0.01). **I** qRT-PCR and WB data showing decrease in both ZEB2 mRNA and protein expressions after transfection with hsa-circ-0004771d. (** vs siNC, P < 0.01). **J** Spearman correlation coefficient analysis showing the relationship between exosomal hsa-circ-0004771 expression in cell culture media and miR-653/ZEB2 expression in CRC cells transfected with si-hsa-circ-0004771, and between miR-653 and ZEB2 in CRC cells transfected with si-hsa-circ-0004771. **K** Transfection of miR-653 mimic into CRC cells decreased the expression of exosomal hsa-circ-0004771 in cell culture media (** vs miR-NC, P < 0.01). **L** qRT-PCR and WB data showing the decrease in both ZEB1 mRNA and protein expression after transfection of CRC cells with miR-653 mimic (** vs miR-NC, P < 0.01). *ZEB2* zinc finger E-box binding homeobox 2, *GAPDH* glyceraldehyde-3-phosphate dehydrogenase, *mRNA* messenger RNA, *si* small interfering, *NC* negative control

We performed morphological analysis of exosomes in NCM460, SW620, and HCT116 cell culture media, by analyzing the surface labeled proteins TSG101 and CD63, by TEM and WB, respectively. The TEM analysis showed that the exosomes from all the above-mentioned cell culture media appeared round or round-like vesicles with a complete cyto-membrane, and the number of exosomes in SW620/HCT116 cell culture media was more than in cell culture media from NCM460 cells (Fig. 5A). Furthermore, exosomes in all above-mentioned cell culture media expressed CD63 and TSG101, but did not express Calnexin. The expression level of CD63 and TSG101 in the exosomes from SW620 and HCT116 cell culture media was significantly higher than that in exosomes from NCM460 cell culture media (P < 0.05) (Fig. 5B). These findings revealed that the exosomes were successfully isolated and identified from NCM460, SW620, and HCT116 cell culture media. Exosome-delivered hsa-circ-0004771 in NCM460, SW620, and HCT116 cell culture media was detected by qRT-PCR. We showed that the exosome-delivered hsa-circ-0004771 expression in SW620/HCT116 cell culture media was elevated as compared to that in NCM460 cell culture media (P < 0.01) (Fig. 5C). Meanwhile, ZEB2 mRNA and protein levels detected by qRT-PCR and WB showed increasing trends in SW620/HCT116 cells as compared to that in NCM460 cell (P < 0.01) (Fig. 5D). It could be seen from the data in Fig. 5E that there was a negative correlation between exosome-delivered hsa-circ-0004771 in SW620/HCT116 cell culture medium and miR-653 level in SW620/HCT116 cell or between miR-653 and ZEB2 at mRNA level in SW620 or HCT116 cell. However, a positive correlation was seen between exosome-delivered hsa-circ-0004771 in SW620/HCT116 cell culture medium and ZEB2 mRNA level in SW620 / HCT116 cells. These results indirectly suggest that exosome-delivered hsa-circ-0004771 in SW620/HCT116 cell culture media may be a regulator of miR-653/ZEB2 in the cells. Furthermore, we successfully established SW620 and HCT116 cells with hsa-circ-0004771-knockdown (P < 0.01) (Fig. 5F). Then, we confirmed that the expression of hsa-circ-0004771 was downregulated in exosomes from cell culture media of hsa-circ-0004771 KD cells, SW620 and HCT116 (P < 0.01) (Fig. 5G). It seems like the exosomes released from SW620 and HCT116 cells into body fluids carry molecules specific to CRC cells. This means that the level of hsa-circ-0004771 in the cells could have a positive impact on the expression of hsa-circ-0004771 in the exosomes obtained from cell culture media. Moreover, the downregulation of hsa-circ-0004771 was associated with an increase in miR-653 level (P < 0.01) (Fig. 5H) and a decrease in ZEB2 mRNA and protein levels in SW620 and HCT116 cells (P < 0.01) (Fig. 5I). Surprisingly, exosome-derived hsa-circ-0004771 in cell culture media from hsa-circ-0004771 KD SW620 and HCT116 cells was negatively associated with miR-653 and positively associated with ZEB2 mRNA level in corresponding cells. In addition, a negative association between miR-653 and ZEB2 mRNA expression was also found in SW620 and HCT116 cells transfected with si-hsa-circ-0004771 (P < 0.05 or P < 0.01) (Fig. 5J). This provides further indirect evidence that hsa-circ-0004771 in cells may regulate miR-653/ZEB2. Additionally, the expression of hsa-circ-0004771 in exosomes released into cell culture media can be positively influenced by cellular hsa-circ-0004771 expression. Also, exosome-derived hsa-circ-0004771 concentration in the cell culture media from miR-653 mimic transfected SW620 and HCT116 cells was downregulated as compared to cells transfected with miR-NC (P < 0.05) (Fig. 5K). As shown in Fig. 5L, ZEB2 mRNA and protein expression were downregulated in SW620 and HCT116 cells transfected with miR-653 mimic as compared to cells transfected with miR-NC (P < 0.01). This could be attributed to the altered expression of miR-653 in the cells, leading to changes in the expression of hsa-circ-0004771 within the cells, subsequently causing corresponding changes in the released exosomes containing hsa-circ-0004771. Besides, miR-653 may regulate ZEB2 in CRC cells.

### Exosome-derived hsa-circ-0004771 in CRC cell culture media mediates 5-FU resistance through targeting miR-653/ZEB2 pathway

**Fig. 6:**
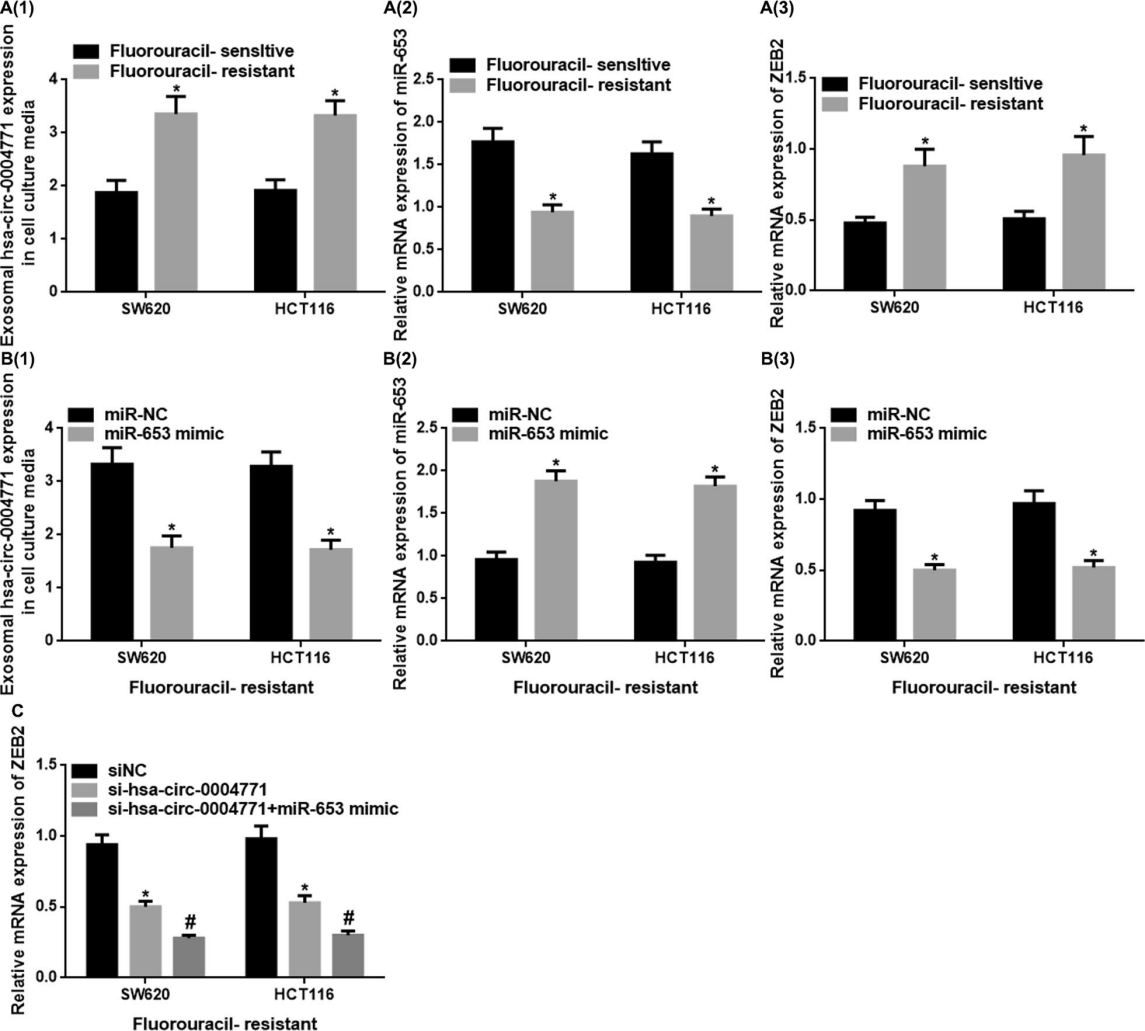
Exosomal hsa-circ-0004771 in CRC cell culture medium mediates resistance to 5-fluorouracil by modulating miR-653/ZEB2 axis. qRT-PCR data showed: **A1** Exosomal hsa-circ-0004771 expression was up-regulated in cell culture media from 5-FU-resistant CRC cells as compared to 5-FU-sensitive CRC cells. **A2** The relative mRNA expression of miR-653 was down-regulated in 5-FU-resistant CRC cells as compared to 5-FU-sensitive CRC cells. **A3** The relative mRNA expression of ZEB2 was up-regulated in 5-FU-resistant CRC cells as compared to 5-FU-sensitive CRC cells (P < 0.05). **B1** Overexpression of miR-653 in 5-FU-resistant CRC cells suppressed the exosomal expression of hsa-circ-0004771 in cell culture media. **B2** miR-653 was overexpressed in 5-FU-resistant CRC cells transfected with miR-653 mimic. **B3** Overexpression of miR-653 in 5-FU-resistant CRC cells down-regulated ZEB2 expression in cells (P < 0.05). **C** The relative mRNA expression of ZEB2 in 5-FU-resistant CRC cells transfected with siNC/si-hsa-circ-0004771/si-hsa-circ-0004771 + miR-653 mimic. (* vs siNC, P < 0.05; # vs si-hsa-circ-0004771, P < 0.05). *mRNA* messenger RNA, *ZEB2* zinc finger E-box binding homeobox 2, *NC* negative control, *si* small interfering

Using qRT-PCR, we discovered that exosome-derived hsa-circ-0004771 was elevated in the cell culture media from 5-FU-resistant SW620/HCT116 cells, miR-653 was downregulated and ZEB2 level was upregulated, as compared to 5-FU-sensitive cells (P < 0.05) (Fig. 6A). This discovery elucidates the involvement of exosomal hsa-circ-0004771, miR-653, and ZEB2 in 5-FU resistance. qRT-PCR was performed to detect the expression of exosome-derived hsa-circ-0004771 in cell culture media; and miR-653 and ZEB2 levels in SW620 and HCT116 cells resistant to 5-FU and transfected with miR-NC or miR-653 mimic, and the results showed that compared to miR-NC group, the expression of exosome-derived hsa-circ-0004771 in the cell culture media transfected with miR-653 mimic was decreased, while miR-653 expression was up-regulated and ZEB2 was down-regulated (P < 0.05) (Fig. 6B). Also, qRT-PCR was used to detect the expression of ZEB2 in 5-FU resistant SW620 and HCT116 cells transfected with siNC/si-hsa-circ-0004771/si-hsa-circ-0004771 + miR-653 mimic. The results showed that ZEB2 was decreased in si-hsa-circ-0004771 group as compared to the siNC group (P < 0.05), while ZEB2 was decreased in si-hsa-circ-0004771 + miR-653 mimic group as compared to si-hsa-circ-0004771 group (P < 0.05) (Fig. 6C). These findings suggest that exosomal hsa-circ-0004771 may be involved in 5-FU resistance, potentially through the regulation of miR-653/ZEB2.

## Discussion

Previous studies have demonstrated the role of drug-resistance in the therapeutic management of various malignant tumors, which challenges the use of different chemotherapies in the clinic [40, 41]. Several reports have shown that the abnormal expression or altered levels of the target molecules, genes (involved in proliferation or apoptosis), and enhanced DNA repair ability were some of the common mechanisms associated with drug resistance [42–44]. In this study, we demonstrated that miR-653 was downregulated in CRC tissues and cells, which associated with the severity and poor prognosis of CRC patients. Also, the overexpression of miR-653 could inhibit cell proliferation, migration and invasion, and promote apoptosis. In addition, serum derived exosomal hsa-circ-0004771 could modulate the resistance of CRC to 5-FU by targeting miR-653/ZEB2 signaling pathway.

Currently, chemotherapy is the first-line treatment for CRC in spite of the limitations due to drug resistance [45]. 5-FU is the preferred first-line chemotherapy for CRC, and it significantly improves survival [46, 47], however, the emergence of drug resistance to 5-FU may reduce its therapeutic effectiveness in the clinic [45]. Increasing data have suggested that exosomes secreted by multiple cell types (including cancer cells) [48, 49] are released into bodily fluids including blood, urine, saliva, etc. [16, 19], which plays diverse functions due to the different cargo it carries (proteins, lipids, DNA and RNA) [50]. Moreover, exosomal RNAs, have been shown to participate in tumorigenesis and progression, epithelial-mesenchymal transition [51], angiogenesis [52], immune reaction and therapeutic resistance [53], and exosomes delivered by bodily fluids could be used as biomarkers for clinical diagnosis [54–56]. It has been reported that serum derived exosomal hsa-circ-0004771 is elevated in CRC patients, and was identified as a biomarker for early diagnosis and prognosis in CRC [24]. In our study, we also found hsa-circ-0004771 to be upregulated in the serum derived exosomes fromCRC patients (Fig. 2A). Previous studies have demonstrated that some factors such as age, sex, BMI, the history of smoking, drinking, and familial inheritance could increase the risk of CRC [57–61]. Therefore, before the comparison of serum derived exosomal hsa-circ-0004771 levels between different groups, we first confirmed that the risk factors mentioned above (the baseline data) were not different between the groups (Table 1), which guaranteed the uniformity between the groups. Moreover, we first identified that exosomal hsa-circ-0004771 in CRC (SW620 and HCT116) cell culture media was also elevated (Fig. 5C), and abnormally elevated exosomal hsa-circ-0004771 in serum from CRC patients or CRC cells culture media was closely associated with 5-FU resistance (Figs. 5B, 6A). In addition, we successfully isolated and identified exosomes from clinical CRC serum samples (Fig. 1) and cell culture media (Fig. 5A, B). Increased exosome release in CRC could be a contributing factor to the increase in hsa-circ-0004771 expression in serum derived exosomes in CRC patients.

For further investigation of the mechanism of serum derived exosomal hsa-circ-0004771 in 5-FU resistance in CRC, we indirectly verified that miR-653 was a target gene of hsa-circ-0004771 (Figs. 3B, 5E, H, J, K), and ZEB2 was a target gene of miR-653 (Fig. 5E, J, L), and miR-653/ZEB2 axis was a pathway targeted by hsa-circ-0004771 (Fig. 5E, I, J) in CRC based on the data from previous studies [25]. In order to further validate that serum derived exosomal hsa-circ-0004771 was involved in the 5-FU resistance by targeting miR-653/ZEB2 pathway, the expression and function, as well as clinical significance of miR-653 in CRC was investigated. We are the first to report that miR-653 was downregulated in CRC tissues (Fig. 3A) and cells (Fig. 4A), and that a lower level of miR-653 reflected higher severity and poor prognosis of CRC patients (Table 2). Going forward, we generated miR-653 overexpressing CRC cells in vitro (Fig. 4B), and showed that the upregulation of miR-653 suppressed CRC cell proliferation (Fig. 4C, D), migration and invasion (Fig. 4E), and induced apoptosis (Fig. 4F). Although very little was found in the literature regarding the expression and function of miR-653 in cancer, there were some published data that reported miR-653 to be downregulated in breast cancer [25], wherein its upregulation caused tumor suppressive effects and suppressed cell proliferation, invasion, metastasis, and tumor growth, and facilitated apoptosis [25–28]. Furthermore, the treatment of CRC with 5-FU induced alterations in cellular functions such as apoptosis, cell cycle, autophagy, respiration, glucose metabolism, etc., and mediated molecular changes including epigenetic changes and microRNA (miR) dysregulations, etc. [14]. These data mean that opposing cell biological behaviors caused by abnormal expression of genes or microRNAs in CRC may participate in promoting 5-FU resistance. In this work, we verified miR-653 was downregulated in 5-FU-resistant SW620/HCT116 CRC cells (Fig. 6A). This finding indicated that miR-653 may be involved in mediating 5-FU resistance in CRC. Meanwhile, we detected that ZEB2 was upregulated in SW620 and HCT116 CRC cells at both mRNA and protein levels (Fig. 5D), this result was consistent with the Li MZ’ report in 2017 [29]. Besides, in Li MZ’ report in 2017, ZEB2 was also overexpressed in clinical CRC tissues samples, and played an oncogenic role, promoting cell proliferation, metastasis, epithelial-mesenchymal transition, tumor growth, and tumor formation [29]. Another interesting finding about ZEB2 in our study was that ZEB2 was overexpressed in 5-FU-resistant SW620/HCT116 CRC cells (Fig. 6A). This finding further supported the conclusion that the upregulation of ZEB2 increased the resistance of CRC to 5-FU [30]. These data suggested that ZEB2 may also be involved in mediating 5-FU resistance in CRC. In the present work, further results showed that (Fig. 6B, C) hsa-circ-0004771 in the exosomes of cell culture media participated in 5-FU resistance by targeting miR-653/ZEB2 pathway in CRC, and there was a clear additive (suppressive) effect between hsa-circ-0004471 and miR-653 mimic. Data from Xie R et al.’s study, online data from circinteractome (https://circinteractome.nia.nih.gov/) and TargetScan (http://www.targetscan.org/) have predicted that some binding sites exist between hsa_circ_0004771 and miR-653 and between miR-653 and ZEB2, and by using dual luciferase reporter gene assay, the target relationships between hsa_circ_0004771 and miR-653, and between miR-653 and ZEB2 were verified. In Xie R et al.’s study, hsa_circ_0004771 functions upstream to inhibit miR-653 expression, while miR-653 acts upstream to downregulate the expression of ZEB2 through binding to its 3'-UTR [25]. So, based on their findings, our study mainly adopted an indirect validation to confirm the above target relationships (Figs. 3B, 5E, H, J, K, L). Therefore, miR-653 may be a target miRNA of hsa_circ_0004771 and ZEB2 may be a target gene of miR-653. Hence, when miR-653 was overexpressed in 5-FU-resistant CRC cells (Fig. 6B2), in order to return the miR-653 expression to normal levels, a higher amount of hsa_circ_0004771 was be needed to bind to miR-653 and reverse its overexpression. This in turn reduced the expression of hsa_circ_0004771 in exosomes released from the 5-FU-resistant CRC cells into the cell culture medium (Fig. 6B1). So, exosomal hsa-circ-0004771 may be positively influenced by cellular hsa-circ-0004771 expression while negatively affected by cellular miR-653 expression. Moreover, the overexpression of miR-653 caused a decrease in the expression of its target gene ZEB2 in the 5-FU-resistant CRC cells (Fig. 6B3). Furthermore, when hsa_circ_0004771 was knocked down in 5-FU-resistant CRC cells, it caused a corresponding decline in ZEB2 expression, and a combination of down-regulation of hsa_circ_0004771 with up-regulation of miR-653 in 5-FU-resistant CRC cells resulted in a lower expression on ZEB2 in the cells (Fig. 6C). This suggests that hsa_circ_0004771 may be involved in 5-FU resistance through targeted regulation of the miR-653/ZEB2 axis. Complementally, previous studies have demonstrated that the expression of hsa-circ-0004771 is elevated in serum-derived exosomes of CRC patients, making it a potential biomarker for early diagnosis and prognosis of CRC [24]. Additionally, exosomes released by CRC cells are involved in chemoresistance [15]. Importantly, ZEB2 is highly expressed in CRC patients’ samples and cell lines [29] and upregulation of ZEB2 has been shown to upregulate resistance to 5-FU treatment in CRC [30]. As it is well known, exosomes, contain cell-specific proteins, lipids, and nucleic acids, making them promising diagnostic markers for diseases [20]. circRNAs are also considered ideal biomarkers in clinical settings due to their resistance to degradation by RNAse (R) in body fluids. Furthermore, the “unique” membrane structure of exosomes provides further stability to circRNAs within them [23, 62]. These exosomes possess biological activity molecules that enable them to exert targeted effects on recipient cells, thereby exhibiting stable functions [20]. Therefore, changes in the expression of hsa_circ_0004771 in exosomes can reflect the change trend of hsa_circ_0004771 expression in CRC cells. It is possible that hsa_circ_0004771 in exosomes may participate in the development of resistance to 5-FU treatment in CRC cells by targeting the miR-653/ZEB2 axis. Serum exosomal hsa-circ-0004771 may a potential markers for predicting 5-FU resistance in CRC patients (Additional file 1: Fig. S1).

In summary, our work was the first to demonstrate that miR-653 was downregulated in human CRC tissues and cells, and that it closely associated with CRC severity and poor prognosis, and that the overexpression of miR-653 was tumor suppressive in CRC, suppressing CRC cell proliferation, migration and invasion, and promoting apoptosis. More importantly, we validated that serum exosomal hsa-circ-0004771 played a vital role in regulating 5-FU resistance in CRC by targeting miR-653/ZEB2 signaling axis. Detection of serum exosomal hsa-circ-0004771 level might be serve as a predictive biomarker for 5-FU resistance in CRC patients in the future Additional file 2.

### Supplementary Information

**Additional file 1: Figure S1.** Effects of miR-653 on CRC cell proliferation. (A, B) Effects of miR-653 on the cell survival of SW620 and HCT116 cells by CCK-8 assay. NC: negative control.

**Additional file 2.** The results of the preliminary experiment about the effects of miR-653 on CRC cell proliferation.

## Data Availability

All the data generated or analyzed in the current study are included in this published article.
