# Supplementary figures and images for: Serum exosomal hsa-circ-0004771 modulates the resistance of colorectal cancer to 5-fluorouracil via regulating miR-653/ZEB2 signaling pathway
Source: Cancer Cell Int. 2023 Oct 16;23:243. doi: 10.1186/s12935-023-03072-9 (PMC10577907; doi:10.1186/s12935-023-03072-9)

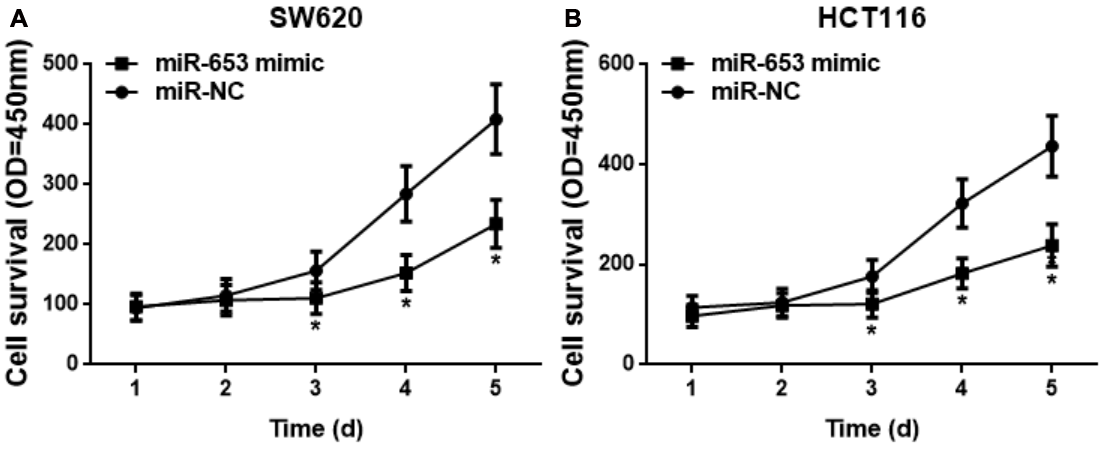

Supplement: Supplementary file 1 — Additional file 1: Figure S1. Effects of miR-653 on CRC cell proliferation. (A, B) Effects of miR-653 on the cell survival of SW620 and HCT116 cells by CCK-8 assay. NC: negative control. [file 12935_2023_3072_MOESM1_ESM.tif]
