# Supplementary material for: Serum exosomal hsa-circ-0004771 modulates the resistance of colorectal cancer to 5-fluorouracil via regulating miR-653/ZEB2 signaling pathway
Source: Cancer Cell Int. 2023 Oct 16;23:243. doi: 10.1186/s12935-023-03072-9 (PMC10577907; doi:10.1186/s12935-023-03072-9)
Supplement: Supplementary file 2 — Additional file 2. The results of the preliminary experiment about the effects of miR-653 on CRC cell proliferation. [file 12935_2023_3072_MOESM2_ESM.docx]

**Supporting information**

**Method**

**Cell Counting Kit‑8 (CCK-8) cell proliferation assay**

CCK-8 assay was performed for determination of cell viability. After transfection of miR-NC/miR-653 mimic into SW620/HCT116 cells for 48 hours, the cells were harvested and seeded into a 96-well plate with a density of 1x10^4^ cells/well. Afterward, 10 µl CCK-8 solution (Beyotime Institute of Biotechnology) was added to each well and incubated in the dark for 2 hours, and then the absorbance at 450 nm was measured using a microplate reader (Bio Tek Instruments). The experiment was repeated three times.

**Statistical analysis**

Statistical analysis was performed using SPSS software (version 20) and the cell survival results were shown as mean ± standard deviation (SD). The comparisons of quantitative data between two groups were conducted using independent-samples t-test and Student’s t-test. Significance levels were set at P < 0.05.

**Result**

**Over-expression of miR-653 inhibited the CRC cells survival**

To determine the cell survival, the cell viability was tested by CCK-8 assay. The results showed that the OD value at 450 nm in miR-653 mimic group was lower than in miR-NC group on day 3 to day 5.

**Figure legend**

**SFigure 1** Effects of miR-653 on CRC cell proliferation. (A, B) Effects of miR-653 on the cell survival of SW620 and HCT116 cells by CCK-8 assay. NC: negative control.
